# Bicuspid Aortic Valve and Thoracic Aortic Aneurysm: Three Patient Populations, Two Disease Phenotypes, and One Shared Genotype

**DOI:** 10.1155/2012/926975

**Published:** 2012-08-27

**Authors:** Robert B. Hinton

**Affiliations:** Division of Cardiology, The Heart Institute, Cincinnati Children's Hospital Medical Center, 240 Albert Sabin Way, Cincinnati, OH 45219, USA

## Abstract

Bicuspid aortic valve (BAV) and thoracic aortic aneurysm (TAA) are two discrete cardiovascular phenotypes characterized by latent progressive disease states. There is a clear association between BAV and TAA; however the nature and extent of this relationship is unclear. There are both distinct and overlapping developmental pathways that have been established to contribute to the formation of the aortic valve and the aortic root, and the mature anatomy of these different tissue types is intimately intertwined. Likewise, human genetics studies have established apparently separate and common contributions to these clinical phenotypes, suggesting complex inheritance and a shared genetic basis and translating 3 patient populations, namely, BAV, TAA, or both, into a common but diverse etiology. A better understanding of the BAV-TAA association will provide an opportunity to leverage molecular information to modify clinical care through more sophisticated diagnostic testing, improved counseling, and ultimately new pharmacologic therapies.

## 1. Bicuspid Aortic Valve Is an Independent Risk Factor for Aortic Valve Disease

Bicuspid aortic valve (BAV, MIM: 109730) is the most common cardiovascular malformation (CVM), occurring in 1-2% of the general population [1], and is a risk factor for aortic valve disease (AVD) [2–5]. AVD (stenosis and/or insufficiency) typically manifests later in life, affecting more than 2% of the population and remains a surgical problem with an increasing frequency of valve replacement procedures performed each year [6–9]. The majority of AVD cases at any age have an underlying BAV, and longitudinal studies in young adults with BAV have shown that >20% ultimately develop AVD requiring intervention [3, 10]. Together, BAV is a congenital malformation and an independent risk factor for AVD. Substantial investigation has established the adverse effects of common comorbid cardiovascular diseases, such as atherosclerosis and hypertension, on the progression of AVD; however, increasing attention on the underlying genetic and developmental processes has identified early mechanisms that incite disease processes.

## 2. Aortic Valve Malformation Is a Spectrum including BAV

**Figure 1:**
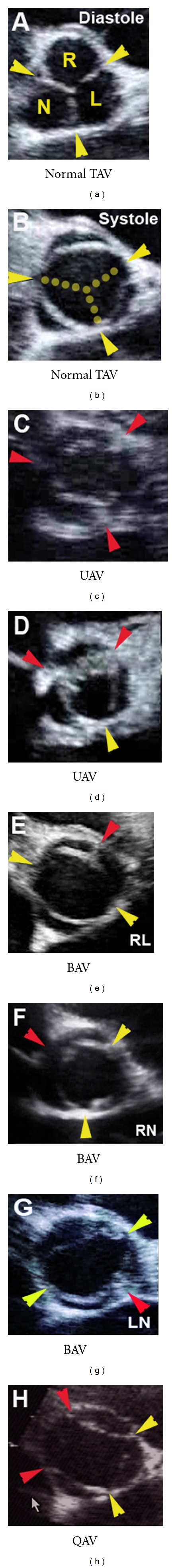
Spectrum of aortic valve malformation. Parasternal short-axis echocardiographic views at the base of the heart showing the aortic valve en face (a–h). Normal tricommissural aortic valve (TAV) morphology is demonstrated in diastole (a) and systole (b). Distinct morphologies are based on fusion patterns of the commissures (dotted lines, (b)) as they relate to the right (R), left (L), and non-(N) coronary sinuses of Valsalva (a). Aortic valve malformation ranges from unicuspid (UAV) to bicuspid (BAV) to a thickened tricuspid (not shown) to quadricuspid (QAV) morphology. Three normal commissures are demonstrated in (a), and normal opening of the commissures results in complete cusp separation to the wall of the aorta at the sinotubular junction (yellow arrowheads). UAV manifests as either partial fusion of all three commissures (red arrowheads, (c)) or complete fusion of both the RN and RL commissures (d). Bicuspid aortic valve (BAV) may manifest as fusion of the RL (e), RN (f), and rarely LN (g) commissures. Rarely, a quadricuspid aortic valve (QAV, (h)) is identified.

Two patterns of BAV morphology are commonly observed: ~70% of cases have fusion of the right and left (RL) coronary cusps with the remainder consisting almost entirely of those with fusion of the right and non-(RN) coronary cusps [3, 5]. Rarely, cases have shown fusion of the left and non-(LN) coronary cusps. It has been proposed that “BAV” actually represents a spectrum of aortic valve malformation ranging from various types of unicuspid aortic valve to quadricuspid aortic valve with the three BAV morphology patterns and a thickened tricommissural aortic valve representing intermediate phenotypes [5] (Figure 1), but it remains unknown why there is uneven frequencies of the different types. Because the relatively rare unicuspid morphology underlies the majority of cases of critical aortic stenosis in the newborn and is associated with hypoplastic left heart syndrome (HLHS), this morphology may have a tendency to develop into aortic atresia and more complex CVM. There have been conflicting reports regarding the association between BAV morphology and AVD [10, 11]. Fernandes et al. identified an association between RN BAV and AVD in a pediatric population, while Tzemos et al. found no association in an adult population [10, 11]. A National Heart Lung and Blood Institute Working Group on AVD recently identified the need to identify “clinical risk factors for the distinct phases of initiation and progression of AVD” [12]. Exploring AVD in a pediatric population allows for examination of the disease process free from the confounding effects of cardiovascular comorbidities. Risk factors for AVD in children are poorly understood [13], but recently Calloway et al. reported that children with RN BAV and adults with RL BAV were more likely to develop AVD [14], suggesting BAV morphology may have predictive value for the time course of AVD.

## 3. Thoracic Aortic Aneurysm Is the Latent Manifestation of Aorta Malformation

**Figure 2:**
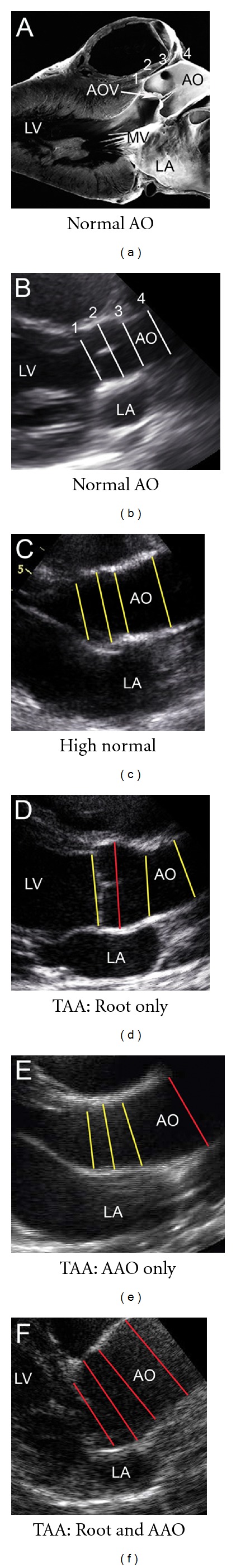
Spectrum of thoracic aortic aneurysm. Pathologic specimen identifies aortic dimensions (1–4) and intimate anatomic relationship of aortic valve and thoracic aorta (a). Parasternal long-axis echocardiographic views of the proximal aorta demonstrating the aortic valve annulus (1), aortic root (2), sinotubular junction (3), and ascending aorta (4) dimensions in normal (b) and discrete patterns of disease (c–f). Some patients are characterized by “high normal” dimensions throughout the proximal aorta (yellow lines, (c)), for example, patients with BAV. TAA may manifest as isolated dilation of the aortic root (red line, (d)), isolated dilation of the ascending aorta (red line, (e)), or dilation of multiple dimensions (red lines, (f)). AOV: aortic valve; AO: aorta; LA: left atrium; LV: left ventricle; MV: mitral valve. Reproduced with permission [22].

Thoracic aortic aneurysm (TAA, MIM%607086) is a subclinical disease state that is typically recognized later in life but can be associated with dissection (TAAD) and sudden death [15–18]. TAA may affect different areas of the proximal aorta, classically isolated to the aortic root, but also including dilation of the ascending aorta only or dilation of both the aortic root and ascending aorta with effacement of the sinotubular junction (Figure 2). TAA was originally attributed to an inflammatory state associated with atherosclerosis, but later was recognized to be a structural defect increasing interest in genetic and developmental contributions to malformation of the aorta wall [19–21]. In this light, aorta malformation is a CVM that is present at birth (even if the aorta dimensions are normal) and predisposes the individual to progressive aortic dilation (TAA) and therefore is a risk factor for dissection.

## 4. The Nature and Extent of the BAV-TAA Association Is Unclear

**Figure 3:**
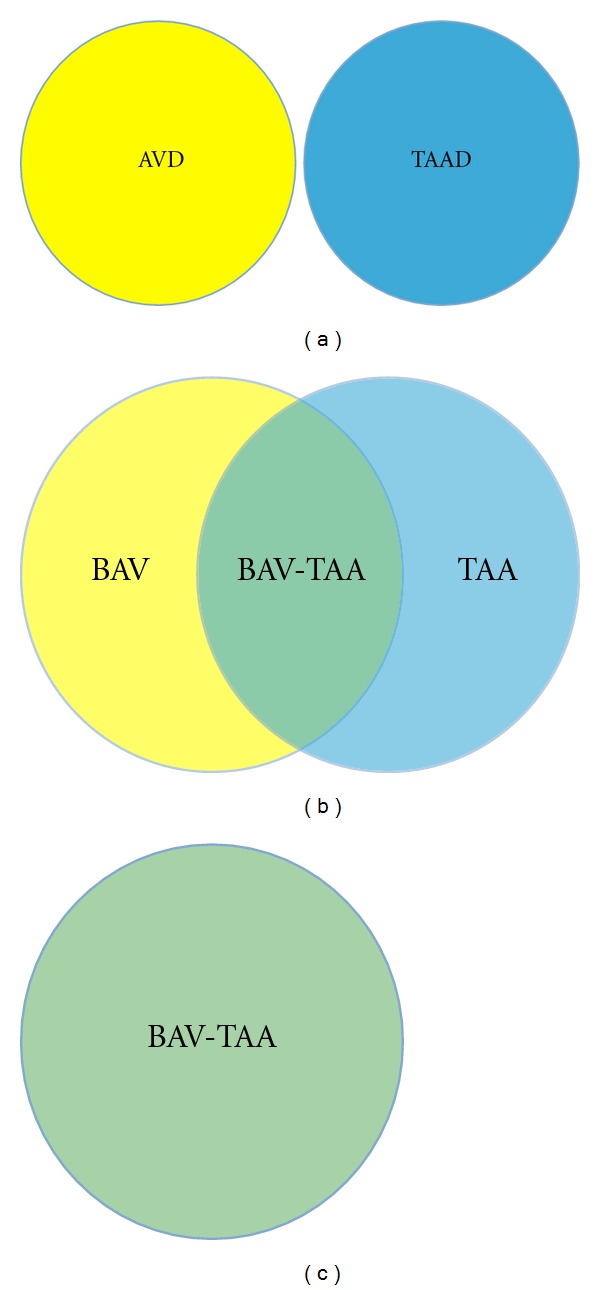
Potential relationships between BAV and TAA. From a disease standpoint, AVD and thoracic dissection (TAAD) represent distinct entities that affect different tissue types (a). However, in light of the clinical association between the respective endophenotypes BAV and TAA, there are 3 patient populations, namely, those with BAV (with or without AVD), those with TAA, and those with BAV and TAA (b). From a genetic and developmental perspective, there is increasing evidence of etiologic overlap, suggesting a shared complex genotype (c).

There are two distinct late-stage disease phenotypes associated with the endophenotypes BAV and TAA, namely AVD and TAAD, and each disease has well-developed consensus guidelines for expectant care [7, 18]. However, there are three patient populations (BAV with or without AVD, TAA with or without TAAD, and both BAV and TAA) that have distinct clinical approaches. From an etiologic standpoint, we know that the vast majority of BAV patients have increased aorta dimensions and approximately 20% have overt TAA that requires surgical repair [23–25], suggesting these processes are not isolated in any patient whether the aorta malformation is evident or not (Figure 3).

Because both malformations are silent and both disease states are latent and because clinical studies including human genetics studies tend to focus on one phenotype, reported frequencies probably underestimate the incidence of the combined phenotype (BAV-TAA). In addition, patients with BAV have degenerative changes in both the media of the ascending aorta and the main pulmonary artery, including diminished and fragmented elastic fibers and smooth muscle cell abnormalities, consistent with a developmental origin [26, 27]. It has been postulated that deficiency of extracellular matrix (ECM) proteins such as elastic fiber components (elastin, fibrillin, emilin, etc.) in BAV patients may activate matrix metalloproteinases (MMPs), leading to maladaptive ECM remodeling and ultimately TAA [28–30]. On the other hand, computational fluid dynamics in the proximal aorta of functional BAV patients demonstrate that asymmetric blood flow patterns may contribute to aneurysm formation, suggesting an acquired disease process in the context of ostensibly normal aorta tissue [31, 32]. It remains unclear if altered hemodynamics represents an independent cause or a secondary exacerbating factor only, for example, AVD or systemic hypertension in TAA. Taken together, malformation of both the aortic valve and aorta is underestimated and may reflect a single disease process (Figure 3). Collectively, previous work supports the idea that BAV and TAA have overlapping genetic etiologies (cause) and shared disease mechanisms (pathogenesis).

## 5. Evidence in Favor of a Single BAV-TAA Malformation

Classifying CVM is challenging. Emphasizing studies designed to elucidate etiology, the National Birth Defect Prevention Study (NBDPS) developed a comprehensive classification system for CVM [33–35]. Botto et al. recognized BAV and TAA as CVMs, but excluded them from consideration because they are not well ascertained at birth, underscoring the difficulty of establishing the presence of silent endophenotypes. CVM, also known as congenital heart disease, represents a broad spectrum of heart defects that are present at birth, often requiring neonatal surgery, and is generally thought to be a pediatric problem. Some CVMs do not manifest as overt disease until adulthood, creating the impression of an acquired disease. There is growing evidence that adult-onset cardiovascular diseases have necessary genetic and developmental components [36, 37], including AVD and TAAD.

BAV is present at birth and may or may not be associated with AVD. Previous studies have identified an association between BAV and a wide variety of other seemingly unrelated CVMs, particularly coarctation of the aorta, ventricular septal defect, and HLHS [38–40]. While it is tempting to think of CVMs as simple (one defect, one affected tissue type), many CVMs affect multiple tissue types, including both valve and aorta. For example, HLHS is a severe CVM characterized by abnormalities of the left-sided valves (aortic and mitral), left ventricular myocardium, and proximal aorta. HLHS may be viewed primarily as valve disease, aortopathy, or cardiomyopathy. BAV is present in 38% of HLHS cases without aortic atresia (the rest having unicuspid aortic valve), and the thoracic aorta is universally affected [40]. Turner syndrome, or X monosomy, is characterized by a spectrum of CVMs, including BAV, HLHS, and TAA [41–43], suggesting a shared genetic predisposition to these phenotypes.

## 6. The Mature Aortic Root Is Composed of Valve and Artery Tissue

Valve anatomy is complex [44–46]. The aortic valve is semilunar in shape and separates the left ventricle from the aorta to promote forward blood flow. Semilunar valves have cusps that are highly organized cell-matrix structures, consisting of fibrosa, spongiosa, and ventricularis layers. The substantial fibrosa layer is composed of fibrillar collagens, while the thin ventricularis layer is composed of elastic fibers and the intervening spongiosa layer is composed primarily of proteoglycans. The distinct shape of the aortic valve creates a unique self-contained support structure within the arterial roots [47, 48]. Like the valve, the aorta is organized in three layers, consisting of the intima, media, and adventitia. The outer layer is made up of collagens and vessels (adventitia), the substantial middle layer is made up of elastic fibers and smooth muscle cells (media), and the thin inner layer is made up of an endothelial lining (intima/subintima). In contrast to the aorta, the aortic root is made up of the fibrous valve annulus region, at the junction between the valve cusp and aortic wall, and the arterial tissue within the sinuses of Valsalva [49–52]. Because the arterial tissue in the aortic root is interrupted by valve tissue extending from the ventriculoarterial junction to the sinotubular junction, there are no lamellae in the aortic root.

**Figure 4:**
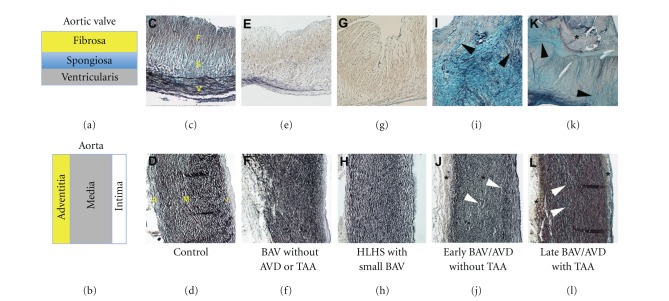
Cardiovascular phenotypes related to malformations of the aortic valve and thoracic aorta. Trilaminar ECM organization of the normal aortic valve (a,c) is characterized by cusps organized into Fibrosa (F), Spongiosa (S), and Ventricularis (V) layers, while the normal proximal aorta (B,D) is characterized by Adventitia (A), Media (M), and Intima (I) layers. Histopathology of a functional BAV, that is a malformed valve without disease, demonstrates preserved ECM organization and normal morphometrics in both the valve (e) and aorta (f). Similarly, the small bicuspid aortic valve of a patient with hypoplastic left heart syndrome, a severe form of aortic valve malformation, shows normal trilaminar ECM organization and morphometrics (g,h). However, BAV with AVD in a younger patient (I) shows ECM disorganization (black arrowheads), in both the affected valve (i) and the aorta with normal dimensions (j). In an older patient with BAV-TAA, there is advanced AVD characterized by marked valve thickening, ECM disorganization (black arrowheads) and calcification (asterisk) and TAA characterized by subintimal hyperplasia, elastic fiber fragmentation, proteoglycan accumulation (white arrowheads), and adventitial fibrosis (asterisk).

Histopathology from both syndromic and nonsyndromic (either familial or isolated) TAA cases has shown similar findings, suggesting common pathogenesis [53, 54]. Studies focusing on human tissue from patients with either aortopathy or valve disease are limited to end-stage specimens that may be confounded by multiple secondary disease processes [30, 55–57]. We have reported a strategy to study mechanisms specific to early and late disease processes by comparing pediatric to adult AVD specimens [58, 59]. Here, we extend these observations and present aortic valve and aorta histopathology from patients with related CVMs, including functional BAV, HLHS, early BAV/AVD without TAA, and late BAV/AVD with TAA (Figure 4).

## 7. Clinical Patterns of Aortic Valve and Aorta Malformation

Interestingly, a functional BAV, that is a malformed valve without stenosis or insufficiency, from an infant that died of noncardiac causes has normal trilaminar architecture and normal valve thickness. Both the aortic root and the ascending aorta (not shown) demonstrate subtle elastic fiber fragmentation, consistent with concomitant aorta malformation, but maintained normal aortic dimensions as measured by echocardiography. Similarly, the aortic valve from a patient with HLHS has a small BAV with normal trilaminar ECM organization. This aorta is small in caliber, but also has subtle elastic fiber fragmentation elongating the normally wavy appearance. A young patient with AVD and an underlying BAV demonstrates valve cusp thickening with marked ECM disorganization but no calcification, while the older patient with AVD and a BAV shows more advanced cell-matrix abnormalities and calcific nodules. The aorta from the young patient with BAV/AVD shows the same characteristic abnormalities and the older patient, consistent with aorta malformation in the absence of overt TAA (Figure 4), but the older patient demonstrates subintimal hypertrophy in addition to these findings, consistent with coexistent inflammatory processes. Taken together, these results demonstrate surprising observations about tissue architecture in different clinical contexts.

In humans, abnormal histopathology is presumably present at birth albeit subtle as shown. Targeted mutagenesis mouse models are power tools to examine mechanisms of human disease [60], and in this regard specific models are instructive. The eNOS-deficient mouse [61] is a model of BAV, and the ACTA2-deficient mouse [62] is a model of aorta malformation and therefore can provide a means to define the pathways and timeline involved in malformation becoming disease. Curiously, the description of the eNOS mutant mouse only superficially examined the aorta, and the description of the ACTA2 mutant mouse did not report observations pertaining to the aortic valve. Studies are needed that explore the regulation of tissue development and homeostasis in both aortic valve and aorta tissue in mutant mice like these that hypothetically affect both tissues and model the BAV-TAA association.

## 8. The Developmental Basis of Both Valves and Arteries Involves Both Neural Crest and Endothelial Cell Progenitors

Numerous developmental signaling pathways have been identified as critical for both valve and aorta disease processes [37, 63–65]. During cardiogenesis, outflow tract development includes aortopulmonary septation and valve formation to produce the semilunar valves and two discrete great arteries (which may explain in part why TAA patients can manifest dilated main pulmonary arteries as well). The first sign of aortic valve development is the formation of endocardial cushions, which is initiated by an endothelial to mesenchymal transformation [66]. Valve cusp formation is characterized by remodeling of the ECM into layers through an elongation process entailing cell proliferation and matrix expansion and remodeling to form the mature valves (reviewed in [45]). The majority of cells that form the semilunar valve cusps originate from endothelial derived cells [67, 68], while the majority of smooth muscle cells that make up the bulk of the proximal aorta are derived from neural crest lineages [64, 69]. In addition, neural crest cells contribute to valve development [70, 71], and endothelial cells are necessary for vascular development [72], consistent with critical functions for both cell populations in both tissue types.

Several developmentally important signaling pathways have critical functions in valve and aorta development, including Wnt, TGF-beta, and Notch signaling. TGF-beta and Notch pathways are particularly compelling in the context of human genetics findings (see below). In the fibrillin-1 mouse model of Marfan syndrome that recapitulates the TAA phenotype, increased TGF-beta signaling has been shown to have a major role in pathogenesis [73], and Losartan, an angiotensin receptor blocker (ARB) that also antagonizes TGF-beta, rescues TAA [74]. These findings have been replicated in a small human cohort and consequently have been the basis of a clinical trial coordinated by the Pediatric Heart Network [75, 76]. Taken together, the molecular effects of an established drug that modulates a latent disease phenotype demonstrates the potential of targeting developmental pathways to identify new therapies.

## 9. Major Genetic Factors Underlie Both BAV and TAA

BAV is heritable and has been shown to be genetically heterogeneous [77, 78]. BAV is associated with Turner syndrome, and mutations in *NOTCH1* have been identified in a small proportion of nonsyndromic AVD patients with BAV [79]. TAA is associated with known connective tissue disorders, for example, Marfan syndrome (OMIM: 154700), and genetic causes of nonsyndromic (familial or isolated) cases of TAA, including mutations in the genes *ACTA2*, *MYH11*, *TGFBR2*, have been established [80–82]. Interestingly, both Marfan syndrome (caused by *FBN1* mutations [83]) and familial TAA caused by *ACTA2* mutations are associated with aortic valve malformation [53, 80]. The prevailing view for a long time was that the manifestation of TAA in patients with BAV was secondary due to hemodynamic alterations associated with AVD [16, 84]; however, the TAA-BAV association persists even when the BAV is functionally normal [23, 85–87].

The BAV-TAA association led to the postulation of a common underlying defect [88]. Subsequently, population studies identified that there is an increased prevalence of TAA in BAV versus non-BAV individuals [89–92]. Previous studies have supported a strong underlying genetic basis for isolated BAV and isolated TAA, including family-based studies that have identified numerous loci for each phenotype that do not overlap for the most part [78, 93–97]. One locus of interest is chromosome 10q23, which is a locus for BAV and TAA, and is the genomic location of *ACTA2*. Limited overlap may be misleading in the context of complex inheritance. For example, a large cohort of BAV families did not identify chromosome 9q34, the location of *NOTCH1*,  missense mutations which cause BAV and calcific AVD [78, 79]. Since Garg et al identified identified pathogenic mutations in patients with BAV, additional studies have identified novel mutations in patients with BAV and TAA [79, 98]. NOTCH1 is an intriguing biological candidate gene. In animal systems, Notch loss of function recapitulates the AVD phenotype and actively regulates the maladaptive development of associated calcification, further supporting a mechanistic role [99, 100]. Loscalzo et al. describe 13 families that segregate both BAV and TAA, typically in multiple generations with approximately one-half of affected individuals having both BAV and TAA [101]. Interestingly, nearly one quarter of families had individuals affected with left-sided lesions, including aortic coarctation and HLHS. Taken together, there are numerous challenges studying these phenotypes, and unraveling the shared genetic basis will require deep phenotyping efforts [102].

## 10. BAV-TAA Association Characterized by Complex Inheritance

**Figure 5:**
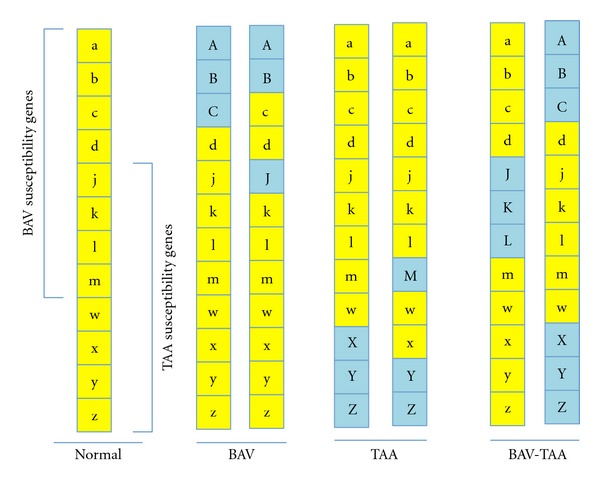
Hypothetical model of shared complex genotype in BAV-TAA. Multiple susceptibility genes exist for both BAV and TAA, and some of these genes are common to both phenotypes (yellow letters, Normal). An unaffected genotype might have 8 BAV susceptibility genes (a–d, j–m) and 8 TAA susceptibility genes (j–m, w–z), 4 in common (j–m). If the manifestation of each phenotype is dependent on a liability threshold of predisposing variants, for example, greater than or equal to 3 variants, then there are multiple ways in which an individual may realize an affected status (BAV, TAA, BAV-TAA), and the specific pattern of variants may contribute to phenotypic variability. Importantly, this model does not take into account the likely importance of additional insults (epigenetic modifiers, environmental factors) that may be necessary for phenotype manifestation.

There is substantial evidence to support the notion that neither BAV nor TAA is single-gene defects. Both phenotypes are characterized by genetic heterogeneity, variable expressivity, and reduced penetrance, consistent with complex inheritance. Pedigree and segregation analyses have consistently identified complex inheritance underlying BAV [77, 101, 103, 104]. Interestingly, an established hamster model of BAV also shows the same characteristics of complex inheritance [105, 106]. Polygenic conditions are characterized by a fixed number of susceptibility genes and a liability threshold (hypothetical model shown in Figure 5). In general, the importance of genetic modifiers and epigenetics is rapidly emerging, but little is known about these factors in the context of BAV or TAA. Different BAV morphologies and different patterns of TAA (Figures 1 and 2) may reflect different combinations of shared genetic variants that carry different clinical risks beyond BAV and TAA, for example, valve disease, aortic dissection, or associated CVM. It has been shown, for example, that RN BAV morphology is associated with a higher risk of developing valve disease, while RL BAV morphology is associated with a higher risk of the associated CVM aortic coarctation, one form of aortopathy [11]. Together, patterns of predisposing genetic variants may translate to variations in clinical disease states, suggesting major modifiers contribute to both phenotypes. Identifying these patterns may impact care, for example, by facilitating the ability to consistently predict natural history [85, 87].

## 11. Genotype Definition and Phenotype Stratification for BAV-TAA Have Significant Clinical Implications

Genetic testing will play an increasing role in the clinical management of BAV-TAA patients. Genotype definition and phenotype stratification  will significantly impact counseling (prognosis, recurrence risk), timing and choice of medication, and surgical disposition (timing and approach). Ultimately, genotype definition may be able to identify those patients with either BAV or TAA that are at risk (or not at risk) of developing the other lesion, impacting clinical management decisions. Screening guidelines for TAA now incorporate genetic information, as do the new Ghent criteria for Marfan syndrome [18, 107], and as more is learned about the genetic basis of BAV, the yield of clinical genetic testing will be sufficient to warrant testing. However, as the genotypes associated with these phenotypes are defined, there may be a need to expand individual Consensus Guidelines for BAV and TAA to include full consideration of the other phenotype or create separate BAV-TAA specific guidelines to reconcile complementary considerations [7, 18].

While there is a clear benefit to lowering blood pressure, molecular developmental insights have elegantly demonstrated the potential of molecular signaling in the tissue to modify disease. Increased understanding of pathogenesis will identify both new therapeutic targets and different groups of patients that will benefit from different therapies. However, both BAV/AVD and TAA/D remain essentially surgical problems; therefore surgical considerations heavily influence clinical management. The pulmonary artery dimension is increased in BAV patients [87, 100], consistent with previously reported histopathologic abnormalities in the pulmonary artery of BAV patients [26]. This may be clinically relevant in BAV patients who require aortic valve replacement and may be candidates for the Ross procedure (autologous pulmonary valve placed in the aortic position). More commonly, some patients with apparently isolated AVD or TAAD undergoing surgical repair may be at risk for subsequently developing the other lesion, for example, the AVD patient who undergoes aortic valve replacement and later develops TAA requiring additional surgical intervention. McKellar et al. recently described aorta complications in 1,286 AVR patients with a median 12-year followup and reported that 10% demonstrate progressive aortic enlargement and only a small minority of these lead to dissection or require further surgery, suggesting prophylactic replacement of the aorta would be warranted in a small subset of patients [108]. The ability to identify which patients are at risk before the first surgery would substantially impact clinical decision making, including for example, a selective approach to combined valve and aorta replacement.

Finally, genotype phenotype information will have important implications for clinical surveillance. For example, current recommendations for functional BAV patients include screening echocardiograms every 5 years [7]. Recently it was shown that surveillance may be modified by morphology such that pediatric patients with RN morphology are screened every 2 years because they are at higher risk of developing new AVD, while individuals with RL BAV could be monitored less aggressively in early childhood as the risk of having AVD at this time is relatively low [14]. Family members of BAV patients may be at risk for TAA (even if they do not have BAV), and family members of TAA patients may be at risk for BAV, underscoring the importance of monitoring for both diseases. In summary, genetic and developmental research advances and future directions (Table 1) promise to continue to provide opportunities for improved care for patients with these important disease processes.

## Figures and Tables

**Table 1:** Summary of concepts and future directions.

(i) Malformation of the aortic valve and aorta shares underlying mechanisms.	
(ii) The complex inheritance underlying BAV and TAA may represent more overlap than previously considered.	
(iii) Genetic testing will play an increasing role in the clinical management of these patients.	
(iv) Future guidelines should consider reconciling complementary considerations by combining recommendations for valve disease and aortopathy.	
(v) Research efforts combining family-based studies with whole-genome exome sequencing promises to help define nonsyndromic BAV-TAA causes.	
(vi) Research efforts studying animal models with both AVD and TAA are necessary to advance mechanistic insights and identify new therapies.	

## Abbreviations

AVD: Aortic valve disease;
BAV: Bicuspid aortic valve;
CVM: Cardiovascular malformation;
HLHS: Hypoplastic left heart syndrome;
TAA: Thoracic aortic aneurysm;
TAAD: Thoracic aortic aneurysm with dissection.
